# The threat of pollutants mixtures on freshwater fishes in Sri Lankan lotic ecosystems under changing climate: a review of current status and future research perspective

**DOI:** 10.3389/fphys.2026.1747210

**Published:** 2026-03-25

**Authors:** W. A. A. N. Wickramasinghe, K. M. S. Ruvinda

**Affiliations:** Department of Zoology and Environmental Management, Faculty of Science, University of Kelaniya, Kelaniya, Sri Lanka

**Keywords:** biodiversity conservation, biomarker, cocktail effect, riverine ecosystem, xenobiotics, biological monitoring, legislative reforms

## Abstract

Multiple point and nonpoint sources add complex mixtures of pollutants that may pose detrimental impacts on freshwater fish. These pollutants include metallic and nonmetallic inorganic ions and an array of organic compounds. Climate-related scenarios and a mixture of contaminants entering riverine ecosystems have impacted many endemic freshwater fish species in Sri Lanka. The present research aims to identify morphological, physiological, and behavioral changes upon exposure to xenobiotics and to predict the influence of climate on these fishes. We discussed the biomarker responses of feral fish, combined with their physicochemical characterization. Moreover, the discussion emphasized empirical evidence from controlled laboratory experiments. Together, these elements were used to interpret the possible future impacts of climate change on the fish in lotic ecosystems. The effects of nanoparticles, microplastics, pharmaceuticals, and endocrine disruptors, and their interplay with climate-related physicochemical variation, have been identified as a research gap. The primary research directions for the future include establishing multi-stressor experimental frameworks that integrate a mixture of xenobiotic exposures of indigenous fishes. The development of standardized biological monitoring protocols that simulate real-world conditions in lotic ecosystems is crucial. Introduction of scientific, evidence-based, robust, and urgent legislative reforms to regulate cumulative pollution may provide a strong legal framework to prevent devastating impacts on freshwater fishes in lotic ecosystems in Sri Lanka.

## 1 Introduction

Freshwater lotic ecosystems are among the most ecologically significant and biodiverse habitats on Earth. They not only play a vital role in maintaining ecological balance and supporting freshwater aquatic life but also provide essential services to human communities that rely on them. Sri Lanka is an island that contains freshwater lotic ecosystems with rich biodiversity and ecological sensitivity (Wimalasena et al., 2024). One hundred three river basins form a dendritic pattern, originating from the central hills. Lotic riverine systems harbor diverse ichthyological diversity, along with several other freshwater habitats, such as tanks, floodplains, pools, villus, and paddy fields (Goonatilake et al., 2020; Nuwanka and Gunathilaka, 2023). The diversity of freshwater fish species is noteworthy, indicating that three-quarters of the identified freshwater fish species are endemic to the country (Senanayake and Moyle, 1982; Pethiyagoda, 1994; De Silva and Silva, 1994; Goonatilake, 2012; Surasinghe et al., 2019; Goonatilake et al., 2020).

Over 9,000 industries are known to be contributing significantly to freshwater contamination in Sri Lanka (Sumaiya, 2024). As an example, according to the ‘Natural Resource Profile of the Kelani River Basin’ published by the IUCN (Goonatilake et al., 2016), there are three types of industries that can be identified in Sri Lanka as type A, B, and C. The amended regulations incorporate a fourth industrial category, referred to as Type D (CEA, 2022). These types are differentiated not only by their environmental impact but also by the jurisdictional authority governing their EPL licensing and compliance enforcement (CEA, 2022). Around 73% of all industries distributed across 20 sub-basins of the Kelani River were categorized into Type A and B industries, confirming the Kelani River as the most polluted lotic freshwater body in Sri Lanka. The major pollutants released from these industries include industrial chemicals and heavy metals (Nuwanka and Gunathilaka, 2023). The intake of xenobiotics into freshwater ecosystems from agricultural waste, industrial discharge, hospital discharge, urban runoff, and inadequate sanitation is a growing concern (Pathiratne and Hemachandra, 2010). Long-term ecological risks are posed due to the accumulation of these contaminants in sediments at higher concentrations—up to a thousand times those found in fish tissue—and they persist in food webs (Dayananda and Liyanage, 2021). Freshwater ecosystems face adverse impacts due to various direct and indirect human activities, resulting in the degradation of water quality and alteration of natural habitats. Despite the vibrant role these ecosystems play in sustaining biodiversity, they face increasing pressures, with climate change further exacerbating existing threats.

Climate change significantly contributes to the amplification of the toxicity of pollutants in freshwater ecosystems (Sunardi and Wiegleb, 2016). Due to the high specific heat capacity of water, increased global temperatures do not directly elevate the temperature of water (Brewer and Peltzer, 2019). However, they still increase the metabolic rates of aquatic organisms, thereby enhancing their uptake of pollutants (Sokolova and Lannig, 2008). Hydrological changes, such as intense rainfall or increased concentration during low-flow periods, can either increase mobilization or concentrate contaminants, thereby increasing the potential toxicity and availability of xenobiotics in freshwater ecosystems (Sunardi and Wiegleb, 2016; Bonet et al., 2023). For instance, increased rainfall can increase the mobilization of sediments, thereby amplifying the effects of pollution, as sediments can accumulate contaminants in high concentrations and be released into waterways. If additional pollutants are mixed with a freshwater stream due to the reasons mentioned above, their combined toxic effects, whether synergistic or antagonistic, may surpass the impact of individual compounds. A meta-analysis of multiple-stressor research on freshwater fish recommends investigating impacts of multiple stressors on endangered fish species, as well as all fish life stages in real ecosystems, over long durations (Lange et al., 2018). This situation complicates risk assessment and management in freshwater lotic ecosystems (Di Poi et al., 2018; Peixoto et al., 2018).

Sri Lanka is vulnerable to climate change and experiences changes in rainfall patterns, thereby experiencing frequent droughts, floods, and extreme weather conditions. These adverse events amplify the impacts of anthropogenic burdens, such as rapid population growth, industrial expansion, and inadequate wastewater infrastructure, which affect water resources and biodiversity (Dananjaya and Wijeratne, 2017). Not only that, but also high freshwater endemism heightens the risk of extinction of species under these stressors (Nuwanka and Gunathilaka, 2023; Sumaiya, 2024). Likewise, the effect of common xenobiotics in Sri Lankan freshwater ecosystems, viz, heavy metals, agrochemicals, pharmaceuticals, and industrial effluents, accumulate in the sediments and aquatic organisms, and further magnify the persistence and toxicity by the climate-driven changes in temperature and hydrology (Borgå et al., 2022; He et al., 2025; Shrivastava et al., 2025). Ultimately, these collaborative impacts contribute to the physiological stress of aquatic organisms, thereby contributing to the decline of sensitive freshwater fish species across Sri Lankan freshwater lotic ecosystems.

There is a significant gap in understanding the interplay between climate change and pollution impacts on fish, due to insufficient research on the topic, particularly in Sri Lanka’s lotic ecosystems (Surasinghe et al., 2019). Consequently, it limits the ability to predict and mitigate the effects of these stressors on fish populations in tropical ecosystems. There is a lack of systematic investigation into the physiological and histopathological consequences of heavy metal exposure within Sri Lanka’s inland freshwater lotic ecosystems (Ruvinda and Pathiratne, 2020). This knowledge is vital for developing effective conservation strategies and pollution management practices. The endocrine-disrupting potential of pollutants has been scarcely investigated, particularly regarding the effects of compounds like Bisphenol-A (BPA) and Bisphenol-S (BPS) on native fish species (Chandran et al., 2025; Matharage et al., 2024). This gap is critical as endocrine disruption can lead to altered reproductive and developmental outcomes in fish (Matharage et al., 2024; Biswas et al., 2021). A plethora of research in river basins has documented how multiple pesticides, fertilizers, and chemical pollutants lead to biological effects, including increased fish mortalities and/or long-term health impairments. Current studies primarily focus on bioaccumulation and health risks, rather than experimental assessments of combined effects, leaving a gap in understanding the broader ecological impacts of pollution on fish communities in lotic ecosystems (Segner et al., 2014). Limited research exists on the combined toxicological effects of pollutant mixtures, particularly how they interact synergistically or antagonistically in local fish species under climate scenarios. Understanding these interactions is essential for assessing the overall health risks to aquatic life. This unresolved issue hinders effective policy-making and environmental protection efforts. Given these overlapping pressures, it is crucial to develop a comprehensive understanding of how xenobiotic contamination and climate change interact. As mentioned, the combined effects can alter species composition, degrade habitat quality, and threaten ecosystem stability, ultimately putting the country’s unique freshwater biodiversity at risk. Therefore, this review examines how a combination of xenobiotics and climate-related stressors jointly impacts the health and long-term sustainability of freshwater fish populations, the studies conducted in Sri Lanka to identify these effects, and the necessary actions to conserve the affected freshwater fish populations in the country.

## 2 Pollutants and sources of exposure

### 2.1 Types of pollutants in mixtures

Aquatic ecosystems are contaminated with a variety of organic chemicals such as, petroleum products (Dupuis and Ucan-Marin, 2015; Hassan et al., 2025), polychlorinated biphenyls (PCB) (Matthies et al., 2016; Mishra et al., 2021), organochloride pesticides (OCPs) (Montuori et al., 2016; Ray and Shaju, 2023), polycyclic aromatic hydrocarbons (PAHs) (Zheng et al., 2016; Raudonytė-Svirbutavičienė et al., 2023; Mishra et al., 2021), polychlorinated dibenzofurans (PCDFs), polychlorinated dibenzo-p-dioxins (PCDDs) (Wang et al., 2016), antibiotics (Carvalho and Santos, 2016; He et al., 2016; Madesh et al., 2024), pesticides (Bunzel et al., 2015; Grung et al., 2015; Ray and Shaju, 2023; Madesh et al., 2024) and cyanobacterial toxins (Corbel et al., 2014; Dixit et al., 2017).

Additionally, inorganic chemicals, including fertilizers, nitrates/nitrites, phosphorus/phosphates, cyanides, chlorides, heavy metals and metalloids (As, Cd, Cr, Cu, Hg, Mn, Ni, Pb, Zn), are continuously added to aquatic environments (Naz et al., 2023). Radioactive pollutants in lotic ecosystems, resulting from accidental releases (Wada et al., 2016) and industrial wastewater (Xin et al., 2023), pose a significant challenge to aquatic organisms. Physical factors, such as turbidity and color pigments, also contribute to aquatic pollution to some extent (Bandara et al., 2011; Kimbell and Morrell, 2015; WHO, 2016). Microbial pollutants, including bacterial pathogens, viral pathogens, protozoan pathogens, helminth pathogens, and radiological hazards, have been introduced into aquatic environments primarily through human activities (Jamil, 2001; WHO, 2016).

Elevated levels of microplastics (Ding et al., 2018; D’Avignon et al., 2022; Banaee et al., 2025; Wu et al., 2025; Bhat et al., 2024; Lawan et al., 2025; Narwal et al., 2025), various pharmaceutical chemicals (David et al., 2020; Kock et al., 2023; Carter and Ward, 2024; Rodrigues et al., 2025), and nanomaterials (Kunhikrishnan et al., 2015; Auffan et al., 2020; Shukur et al., 2023; Garg et al., 2025), have been reported in various aquatic habitats in recent years. Several endocrine-disrupting chemicals (EDCs) are now recognized as emerging pollutants in aquatic environments. These include BPA, diethylstilbestrol, estrone, 17α-ethinylestradiol, nonylphenol, and phthalates (Zhou et al., 2019; Celino-Brady et al., 2021). Pollutants are being added to the lotic ecosystem as mixtures in the form of point or nonpoint sources in adjacent areas.

### 2.2 Point and nonpoint sources of aquatic pollution in Sri Lanka

Pollutants enter the aquatic environment through point sources, including hazardous spills, underground storage tanks, chemical storage piles, mine-waste ponds, deep-well waste disposal, industrial or municipal waste outfalls, runoff, leachate from municipal and hazardous waste dumpsites, and septic tanks. Nonpoint sources of freshwater pollution are agricultural activities (irrigation and drainage, pesticide and fertilizer applications, livestock waste), aquaculture activities, urban and industrial runoff, erosion associated with construction, mining and forest harvesting activities, atmospheric deposition, and hydrological modification (dams, diversions, channelization, over pumping of groundwater, siltation) (Loague and Corwin, 2006; Malik et al., 2020; Gunasekara et al., 2025). Sometimes, particular contaminants can be introduced by several sources. For example, ammonia can be added to water through agricultural practices, aquaculture, industrial activities, as well as transportation and wastewater. Meanwhile, there may be links between several activities in the production of contaminants (WHO, 2016).

Freshwater lotic ecosystems in Sri Lanka face significant pollution challenges from both point and nonpoint sources, threatening aquatic biodiversity and ecosystem health. Point-source pollution refers to contaminants discharged from identifiable sources, such as industries and municipalities, and nonpoint source pollution refers to contaminants entering water bodies from diffuse sources rather than a single point of discharge (Surasinghe et al., 2019). Freshwater surface water bodies are most polluted by inadequate management of sewage, industrial effluents, and agricultural runoff (Nuwanka and Gunathilaka, 2023; Quyen et al., 2021). The demand for freshwater has increased due to population growth and industrialization, creating more complex pollution-related scenarios that require an inclusive understanding of pollutant sources, types, and their spatiotemporal variations (Nuwanka and Gunathilaka, 2023).

**FIGURE 1:**
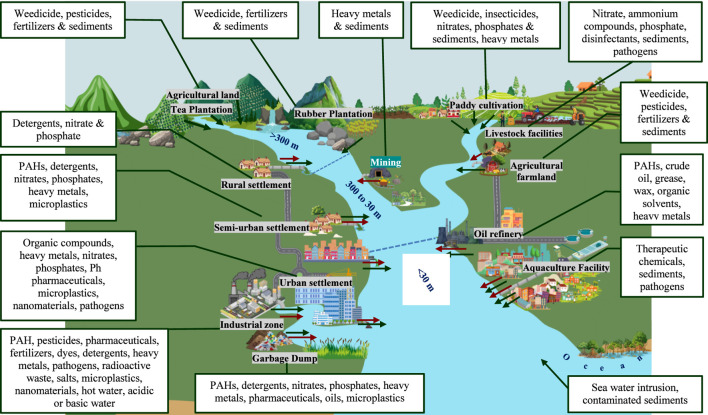
Pollution mixtures discharged by point (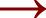) and nonpoint sources (→) into Sri Lankan lotic ecosystem.

Sri Lankan lotic water system flows to the ocean from the central highland (Figure 1). In high-altitude regions (above 300 m), rivers are characterized by steep gradients and high rainfall. The pollution here is primarily linked to nonpoint runoff from tea, rubber, and agricultural lands, as well as to natural processes, such as erosion. Tea and Rubber Plantations are the most significant contributors to freshwater pollution, as they carry fertilizers (nitrogen and phosphorus) and pesticides into tributaries. Land degradation and steep terrain increase sediment load, increasing water turbidity. Point Sources are minimal in this area, but small-scale rubber processing centers discharge effluent directly into streams. Meanwhile, the domestic sewage of sparse settlements often discharges a small number of pollutants (Figure 1).

In mid-elevated areas (30–300 m), freshwater lotic systems enter the transitional plains and begin to diversify the pollution sources (Figure 1). Many point sources of pollution, such as mining, textile industries, chemicals manufacturing and formulating facilities, food processing, and pharmaceutical production discharge waste containing water into lotic ecosystems in Sri Lanka. Factories manufacturing or formulating chemicals release organic and inorganic chemicals such as soaps, detergents, softeners or any other cleansing preparations, fertilizers, pesticides including insecticides, fungicides, and weedicides. Storage facilities of coal, mineral oil, petroleum, as well as vehicle stations, plant and animal oil extraction industries that add oil, wax, and solvents, are considered point sources. Industries involved in the manufacture of polymers or polymer-based products (i.e., polyethene, polyvinyl chloride (PVC), polyurethane, polypropylene, polyester, nylon, polystyrene, resins, fiberglass, or other artificial fibers) and tire retreading or rebuilding industries add microplastics, fibers, and particles. Inorganic nutrient loads are added to lotic habitats through rice mills, hatcheries, poultry farms, piggeries, or cattle/goat farms. Pharmaceuticals, disinfectants, and residues of medical chemicals are primarily added by municipal wastewater and hospital effluents. Additionally, the tea, rubber, and coconut processing industries produce wastewater laden with organic and chemical pollutants, while power plants and mining operations contribute to thermal pollution and the release of heavy metals (Hemachandra and Pathiratne, 2017b; Arachchige et al., 2019; Danthurebandara and Rajapaksha, 2019; Goswami et al., 2022). Collectively, these point sources degrade water quality and encourage bioaccumulation of contaminants in aquatic populations. Semi-urban runoff carrying oil and street litter enters lotic systems, contributing to nonpoint-source pollution. The primary contributor to nonpoint pollution in Sri Lanka is agriculture, particularly the use of fertilizers, herbicides, and pesticides in paddy fields, tea plantations, and vegetable farms, which are washed into water bodies during rainfall (Marambe and Herath, 2020). Urban stormwater runoff and atmospheric deposition are also nonpoint sources of pollution. Oils, grease, plastics, and heavy metals from roads and built environments are contained in stormwater. Combustion by-products and vehicle emissions enter aquatic systems through atmospheric deposition (Weerasundara and Vithanage, 2015; Weerawardhana, 2024). Soil erosion and sedimentation resulting from deforestation, construction, and poor practices also enhance pollutant transport, affecting water clarity and aquatic habitats (Jayawardana and Edirisinghe, 2016).

The lower reaches of streams and rivers (below 30 m) showed a high level of pollution due to increased human activity. Large-scale discharge from densely populated urban areas often enters the river through major canals. Direct discharge of waste oil from vehicle service centers is a significant localized issue. Illegal settlements along the riverbanks and canals generate significant amounts of solid waste, plastics, and untreated human waste that enter the water across a broad area. Saltwater intrusion during the dry season affects water quality at the lower reaches of some major rivers (Figure 1).

Overall, both point and nonpoint sources of pollution contribute to worsening freshwater quality, increasing the bioavailability of toxic compounds, and exacerbating physiological and ecological stress on freshwater fish populations in Sri Lanka’s lotic ecosystems.

## 3 Pollutants and their impacts on freshwater fishes

### 3.1 How fish are exposed to pollution mixtures

**FIGURE 2:**
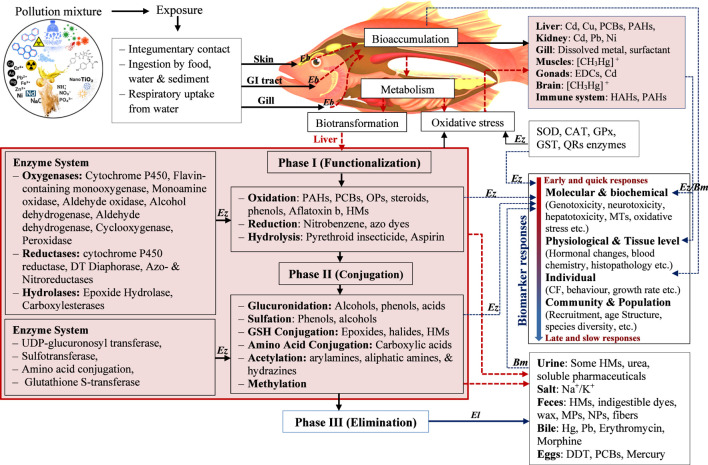
Mechanistic pathways of complex pollutant mixtures in fish body and different levels of biomarkers (*Ez*, Enzyme; *Bm*, Biomolecules; *El*, Elimination; *Eb*, Absorption/intake; 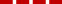
blood circulation, ----Biomarker).

Xenobiotics in the aquatic pollution loads trigger a range of biochemical, physiological, and histopathological responses in fish. The toxic effects of xenobiotics and mixtures depend on biological processes, including absorption, distribution, biotransformation, and elimination. By altering the effective toxicant concentration at the target site over time, these processes influence the whole-organism dose-response relationships (Escher et al., 2011). First, xenobiotic chemicals enter the bloodstream of the organism through absorption (as illustrated in Figure 2). Xenobiotics can be accumulated in fish through direct uptake from water, dermal exposure, or gill uptake, or *via* the ingestion of suspended particles or contaminated food (Erickson et al., 2008; Kleinow et al., 2008; Maurya and Malik, 2016; McKim, 2024; Wu et al., 2025). Branchial Uptake of xenobiotics is the most common route, as large volumes of water move through their gills during respiration. Many water-soluble chemicals or suspended particles come into constant contact with the thin, highly vascularized gill membranes and enter the body through passive diffusion. Pollutants enter the body through dietary uptake when they consume contaminated prey, sediment, or plants. Digested materials are absorbed through the gastrointestinal epithelium. Diary route causes bioaccumulation of toxic components and causes short-term and long-term biophysiological changes in fish (Lee et al., 2024). As fish are immersed in an aquatic medium, dermal uptake is a significant, viable route of toxic pollutants, especially lipid-soluble pollutants (Ray and Shaju, 2023). The dermal route is much more critical for larval fish or embryos, which have thinner, more permeable skin than adult fish (Erickson et al., 2008).

Then the blood circulation distributes the xenobiotics around the body, and they undergo chemical alteration through metabolism or the biotransformation process within the organism’s body, which accelerates their removal through excretion (Flanagan et al., 2020). Xenobiotics tend to be eliminated from the fish’s body when they are bioaccumulated or biologically available (Figure 2). There are two major pathways: either excretion of chemicals in their original form or biotransformation into hydrophilic compounds, which are more readily excreted than the original compound (Newman and Newman, 2014; Ashwath et al., 2023). The liver is the most commonly involved organ in the biotransformation of xenobiotics due to its position, function, and blood supply (Hinton et al., 2017; Topić Popović et al., 2023; Schlenk et al., 2024).

### 3.2 Impacts of pollution mixtures on the fishes

The biological responses to pollution mixtures range from molecular to cellular and physiological responses, as well as behavioral changes in individual organisms, to population, community, and ecosystem function and structure of ecosystems (Van der Oost et al., 2003; Colin et al., 2016; Madesh et al., 2024). Sub-lethal exposure to chemical pollutants can induce pathological conditions and tissue and organ alterations at molecular, cellular, or physiological levels (Lionetto et al., 2019). Some of these disease signs are macroscopic, such as tumors, skeletal malformations, fin erosion, and increased volume of target organs, and can be easily quantified *via* gross visual examination and tissue-whole body mass indices, for instance, Hepato-Somatic Index (Van Dyk et al., 2012; Al-Ghais, 2013; Colin et al., 2016). A first-level screen to identify potential effects and xenobiotic exposure can be accomplished based on measures of the body condition of fish. The most sensitive members of a fish population may be identified by such measures (Omondi et al., 2024). In field studies, one commonly measured morphological parameter is the condition factor (CF), which is used to evaluate the overall health status of fish and is calculated as 100 × [weight (g)/(length (cm))^3^] (Agumassie, 2018; Azrita et al., 2024). CF may provide information about energy reserves; limitation of food or consumption of food of fish is impaired as a result of toxic challenges and environmental stressors (Morado et al., 2017). Determining CF is a simple, non-destructive, and non-invasive method, making it a very attractive biomarker for field biologists (McPherson et al., 2011). Upon exposure to pollution, the metabolic cost of detoxifying the chemical compounds results in poor growth, reduced forage efficiency, and synergetic stress. Such growth and developmental alterations in cells and whole tissue architecture are only detected *via* histopathology.

Traditionally, histopathology has been a descriptive science, with some subjectivity in selection, processing, and examination of the target tissues. However, guidelines are now available to score and report histopathological data of fish (Rey et al., 2020), even following well-established international procedures for organs such as the liver and gonads. Chemical pollution has been associated with a wide range of histological alterations, including inflammatory processes, necrosis, vascular disorders, and the presence of neoplastic cells, reflecting the severity and duration of pollution events. Positive correlations between pollutant levels in pollutants, for example, pharmaceuticals, pesticides, and histological alterations such as neoplastic lesions and testis-ova are reported for several species both in field and laboratory conditions, further supporting the use of histopathological biomarkers as good indicators of aquatic pollution (Van Dyk et al., 2012; Liebel et al., 2013; Jordanova et al., 2016). Other advantages of histopathology include the ease of sample collection, processing, and storage, as well as the ability to assess multiple body systems from the same individual. In addition, histopathological biomarkers (e.g., testis-ova) can identify the type of pollution *via* its mode of action, such as endocrine disruption, although they cannot reveal the toxic identity. Advanced histopathological techniques, such as *in situ* hybridization and immunohistochemistry, can refine these diagnostics; however, they are currently only available for laboratory models, such as zebrafish (*Danio rerio*) (Colin et al., 2016).

The deep-rooted use of micronuclei formation and erythrocytic nuclear abnormalities has served as a reliable and sensitive index of cytogenetic damage (Bolognesi and Hayashi, 2011). Various studies have shown that the peripheral erythrocytes of fish have a high potential of the induction of micronuclei and nuclear abnormalities after exposure to different pollutants under field and laboratory conditions (Al-Sabti and Metcalfe, 1995; Russo et al., 2004; Ossana et al., 2016), genotoxicity evaluation of physical and chemical agents after direct or indirect exposure (Braham et al., 2017). Micronuclei are formed by condensation of chromosomal fragments or whole chromosomes that are not included in the central nucleus following anaphase (Kiraly et al., 2017). Blebbed nuclei presents a relatively small evagination of the nuclear membrane, which contains euchromatin. Evaginations larger than the blebbed nuclei, which could have several lobes, are classified as lobed nuclei. Nuclei with vacuoles and appreciable depth into a nucleus that does not contain nuclear material are categorized as notched nuclei (Braham et al., 2017). Fish in lotic ecosystems exposed to a mixture of pollutants might develop nonspecific histological and cytological alterations that need to be further investigated using biochemical methods.

The next level of toxicological impacts is that the levels of biotransformation enzymes in fish can be inhibited or induced depending on exposure to pollutants (Burkina et al., 2020; Schlenk et al., 2024). The process of biotransformation involves two major phases (Figure 2). Phase I involves functionalization—adding or unmasking reactive functional groups through oxidation, reduction, or hydrolysis (Schlenk et al., 2008). Oxygenases such as Cytochrome P450 and microsomal monooxygenase enzyme system catalyses xenobiotic biotransformation in phase I of the liver in fish (Schlenk et al., 2008; Lavado and Schlenk, 2011; Loerracher and Braunbeck, 2021). Reductases function by adding electrons to molecules under low-oxygen conditions, effectively breaking down complex structures like azo or nitro compounds into simpler primary amines. In hydrolysis step, Epoxide Hydrolase and Carboxylesterases use water to cleave chemical bonds, converting reactive epoxides into stable diols or breaking down esters and amides into their parent acids and alcohols (Schlenk et al., 2008). Phase II includes the conjugation of the xenobiotic parent compound or its metabolite with endogenous ligands (Schlenk et al., 2008; Katagi, 2020). Lots of these enzymes facilitate chemical excretion by adding a polar group. They also play a significant role in homeostasis and in detoxification and clearance of many xenobiotic compounds. Conjugation with glutathione (GSH) and the conjugation of neutrophilic compounds with glucuronic acid are the primary pathways for electrophilic compounds and metabolites (Gwinn et al., 2021; Mahanayak, 2024).

The measurement of a phase I biotransformation enzyme, Cytochrome P4501A (CYP1A) dependent ethoxyresorufin-O-deethylase (EROD) in fish is a sensitive but nonspecific enzymatic probe that can be used to detect highly toxic organic pollutants in aquatic environments, such as PAHs, PCBs, dioxins, pesticides, petroleum products, and drugs (Pathiratne and Hemachandra, 2010; Yılmaz, 2010; Valdehita et al., 2012; Colin et al., 2016; Fabrin et al., 2018). The EROD activity is measured by following the rise in fluorescence of the reaction product resorufin (Vehniäinen et al., 2012). Good connections have been observed between CYP1A protein levels, EROD activity, and research mechanisms of CYP1A-induced toxicity, suggesting that EROD activity not only indicates chemical exposure but also precedes effects at various levels of biological organization (Koglin et al., 2016).

The conjugation of electrophilic phase I metabolites with Glutathione (GSH) is catalyzed by the glutathione S-transferases (GSTs) enzymes. GST plays a critical role in defending against oxidative damage and peroxidative products of DNA and lipids while performing its essential functions in the intracellular transport of heme, bilirubin, and bile acids, as well as the biosynthesis of leukotrienes and prostaglandins (Frank et al., 2011; Mazari et al., 2023). Studies determine the total GST activity using the artificial substrate 1-chloro-2,4-dinitrobenzene (CDNB), which is conjugated by all GST isoforms, except of the q-class enzymes (Aksoy et al., 2016). Since GST plays a crucial role in the detoxification of xenobiotics, inhibition or induction has a significant effect on the metabolism of environmental contaminants (Boušová and Skálová, 2012).

A mixture of pollutants triggers the formation of reactive oxygen species (ROS), causing oxidative damage and stress in fish (Birnie-Gauvin et al., 2017; Chowdhury and Saikia, 2020; Madesh et al., 2024; Formicki et al., 2025). Combining molecular oxygen reduction with energy generation during aerobic metabolism creates the potential of oxidative stress, i.e., damage to critical cellular macromolecules, which may lead to enzyme inactivation, lipid peroxidation, DNA damage, and ultimately, cell death (Regoli and Giuliani, 2014). Major types of free radicals that cause oxidative damage in fishes include superoxide radical (O2•−), hydroxyl radical (OH•), hydroperoxyl radical (HOO•), alkyl peroxyl radical (LOO•), alkoxyl radical (LO•), and nitric oxide (NO•) (Formicki et al., 2025). Hydrogen peroxide (H_2_O_2_), as well as free radicals such as the superoxide free radical (O_2_•^–^) and the hydroxyl radical (OH•), are highly potent oxidants that can create oxidative stress in organisms (Ifeanyi, 2018). Elevated oxidative stress can be indicated by increased levels of superoxide dismutase, catalase, and glutathione peroxidase, as well as lipid peroxidation, in exposed fish (Newman and Newman, 2014; Birnie-Gauvin et al., 2017). The complex physicochemical nature of aquatic ecosystems, along with emerging pollutants such as heavy metals, industrial effluents, pesticides, and nanomaterials, as well as land use changes, together with intrinsic factors like life histories, nutrition, food deprivation, and physical activity, influences the oxidative stress of fish (Birnie-Gauvin et al., 2017; Formicki et al., 2025).

Exposure to endocrine-disrupting chemicals from point or nonpoint sources may affect body weight, cell growth, cell differentiation, biochemical activities (including alterations of protein/enzymes), gene expression, sex determination (Celino-Brady et al., 2021; Dasmahapatra et al., 2023). Fishes captured from the Xiangjiang River, China, *Parabramis pekinensis, Cyprinus carpio* and *Siniperca chuatsi* accumulated higher contents of bisphenol A (BPA), diethylstilbestrol and estrone endocrine disruptors in the liver and muscles (Zhou et al., 2019).

The expected behavior and muscular function of animals depend on cholinesterase (ChE) enzymes. The neurotransmitter acetylcholine breaks down into choline and acetic acid by Cholinesterase (ChEs) enzymes, especially AChE. This hydrolysis is vital for the normal functioning of sensory and neuromuscular systems, as it prevents overstimulation by allowing neurons to return to their resting state after a signal has been sent. Main types of ChE are acetylcholinesterase (AChE), butyrylcholinesterase (BChE), also known as nonspecific esterase or pseudocholinesterase, propionylcholinesterase (PChE), and carboxylesterase (CbE) (Formicki et al., 2025). The responsiveness of BChE, PChE, and CbE to organic and inorganic pollutants and various chemicals significantly varied among fish species. At the same time, these enzyme activities differ depending on the specific tissue being examined (Formicki et al., 2025). In fish, while muscle tissues contain both AChE and BChE, the brain contains only AChE (Miao et al., 2010; Colovic et al., 2013). The exact biological functions of these enzymes are not yet fully understood, but AChE remains the most frequently utilized biochemical indicator for identifying neurotoxicity among the various cholinesterases (Formicki et al., 2025). The inhibition of ChEs is used as a valuable biochemical biomarker upon exposure to many organophosphates (OPs) and carbamate pesticides in organisms. However, this inhibition can lead to constant accumulation of ACh in the synaptic clefts, resulting in nonstop stimulation of nerves, muscles, and glands. It results in characteristic symptoms of poisoning of the organism and, in severe cases, death (Cano-Rocabayera et al., 2022; Aroniadou-anderjaska et al., 2023).

A mixture of pollutants can ameliorate a wide range of ecophysiological changes in fishes. The physiological functioning of fish degraded, ranging from gross clinical signs to deep-rooted neural, immunological, and endocrine disruptions, ultimately leading to reduced growth, reproductive failure, and population collapse (Figure 2). Indigenous ichthyological fauna in Sri Lanka are continuously exposed to a mixture of pollutants, resulting of varying biological impacts.

### 3.3 Pollution impacts on freshwater fishes in Sri Lanka

Freshwater aquatic ecosystems in Sri Lanka are increasingly threatened by anthropogenic pollutants irrespective of their fish endemism (Pethiyagoda, 1994; Surasinghe et al., 2019; Goonatilake, 2024). Research across Sri Lankan freshwater system has demonstrated that fish are exposed to a wide range of contaminants, including polycyclic aromatic hydrocarbons, plasticizers, pharmaceuticals, heavy metals, and agrochemicals (Nuwanka and Gunathilaka, 2023). These biological responses range from molecular to cellular and physiological responses, as well as behavioral changes in individual organisms, to population, community, and the function and structure of ecosystems (Colin et al., 2016). Oxidative stress, genotoxicity, histopathological damage, and neurotoxicity have been evaluated using *Oreochromis niloticus* as a standard model and sentinel species in Sri Lanka (De Silva and Pathiratne, 2023). The consistency of observed responses, particularly in gill and liver tissues, as well as erythrocyte abnormalities, suggests strong biomarker potential for monitoring pollution impacts. Moreover, these chemical stressors likely interact with climate-induced stress factors, such as elevated temperatures and altered oxygen regimes, thereby compounding physiological strain and threatening the survival of sensitive freshwater fish species in Sri Lanka’s lotic ecosystems (Table 1).

**TABLE 1:** Different types of organic, inorganic, and a mixture of pollutants reaching aquatic habitats and their biological impacts on fish inhabiting the freshwater habitats of Sri Lanka.

Pollutant types	Species of fish	Impact on the fish	References
Organic pollutant mixtures			
– PAHs^a^ – Naphthalene – Phenanthrene – Pyrene – Benzo(a)pyrene	– Oreochromis niloticus – Mugil cephalus – Lutjanus russellii – Etroplus suratensis – Oreochromis mossambicus	– SDH^b^, EROD^c^, GST^d^, biliary FACs^e^ ↑ – Muscle ChE^f^ activities↓ – Liver lesions and abnormalities – Gill hyperplasia and liver alterations – Erythrocytes with micronuclei ↑ – Nuclear abnormalities↑ (nuclear buds, binucleated, apoptotic cells)	– Pathiratne and Hemachandra., 2010 – Pathiratne et al., 2010a – Hemachandra and Pathiratne, 2014 – Ranasingha and Pathiratne, 2015
– Petroleum refinery wastewater (PAHs^a^)	– Oreochromis niloticus – Trichogaster pectoralis – Dawkinsia singhala	– Lethargic and sluggish behavior – RBC micronuclei and of nuclear abnormalities↑ (bud, binucleated, notched nuclei)	– Weerakkodige et al., 2021
– Oil- contaminated water (PAHs^a^)	– Oreochromis niloticus	– RBC micronuclei and of nuclear abnormalities ↑ (bud, binuclei, notched nuclei, binucli, fragmented apoptotic cells and altered nuclei)	– Kanthi and Jayaweera, 2021
– Crude oil (arabian light) containing water	– Oreochromis niloticus	– Growth↓ – Erythrocytes with micronuclei↑ – Nuclear abnormalities↑ (nuclear buds, fragmented-apoptotic nuclei, blebbed nuclei, notched nuclei, lobed nuclei, binucleated RBC	– Gunawickrama et al., 2016
Agrochemicals			
– Carbosulfan^g^ – Bispyribac-sodium^h^	– Poecilia reticulata – Aplocheilus parvus	– Liver apoptosis in fish – Gill necrosis – Lamellar fusion – Damaged nuclei in the liver	– Fernando et al., 2018
– Organophosphate^g^ – N-methyl carbamate^g^	– Garra ceylonensis – Devario malabaricus – Rasbora daniconius	– Inhibition of AChE activity (muscle, eye tissue, and brain)	– Sumith et al., 2012
– Paraquat^h^ – Fenthion^g^ – Phenthoate^g^	– Rasbora caverii	– Inhibition AChE^i^ activities – Induced gill histopathology (hyperplasia, hypertrophy and enlargement of secondary lamellae forming club shaped deformities)	– Wijeyaratne and Pathiratne, 2006
Synthetic chemicals			
– BPA^j^	– *Danio rerio* ^k^	– Morality ↑ – Altered growth (significantly high length increment and specific growth rates) – Condition factor ↓ – Female-biased sex ratios – Kidney: Damaged renal tubules, shrinkage of tubules or tubule lumen, degeneration of tubules and hematopoietic tissue, structural distortion – Liver: Lipid accumulation in hepatocytes, structural distortion – Increased ammonia excretion – Increased aggression – Reduced fish swimming speed	– Pathiraja and Rajapaksa, 2019 – Senarath Pathirajage and Rajapaksa, 2024 – Shanika and Rajapaksa, 2024 – Shanika and Rajapaksa, 2025
– BPS^l^	– *Danio rerio* ^k^	– Morality ↑ – Altered growth - significantly high length and weight increment and specific growth rates – Female-biased sex ratios – Increased ammonia excretion – Increased aggression – Reduced fish swimming speed	– Shanika and Rajapaksa, 2024 – Shanika and Rajapaksa, 2025
– Acetaminophen	– Oreochromis niloticus – Danio rerio	– RBC nuclear abnormalities ↑ (bi-nucleated, notched, lobed, blebbed nuclei) – Altered gill histopathology – Behavioral changes: Longer time to detect food, attenuated physical Avoidance responses – Mean ventilation rate↓ – Mean maximum swimming speed ↑ – Mirror-biting frequency ↑ – Ammonia excretion ↑ – Vitellogenin expression ↑	– Dissanayaka et al., 2024 – Ishara et al., 2024 – Rajapaksa and Ishara, 2025
Biological chemical			
– Microcystins	– Oreochromis niloticus	– SDH^b^, EROD^c^, GST^d^, biliary FACs^e^ ↑ – Muscle ChE^f^ activities ↓ – Structural abnormalities in the liver	– Pathiratne et al., 2010b
Inorganic pollutants			
– Cd – Cr – Cu – Pb – Zn – As	– Oreochromis niloticus – Etroplus suratensis – Dawkinsia singhala – Poecilia reticulata	– Mortality ↑ – Brain ChE^f^ activity ↓ – RBC and WBC count ↓ – RBC micronuclei and nuclear abnormalities (nuclear bud, binuclei notched – Histopathologic indices ↑ – Liver lesions	– Ruvinda and Pathiratne, 2018 – Ruvinda and Pathiratne, 2020 – Arambawatta-Lekamge et al., 2021 – Dayananda and Liyanage 2021 – Gayashan et al., 2023
– Raw source water – Water treatment waste – Cd, Cr, Cu, Pb, Zn trace amounts	– Oreochromis niloticus	– RBC micronuclei and nuclear buds ↑ – Total comet scores ↑ – DNA strand breaks	– Hemachandra and Pathiratne, 2017a – Hemachandra and Pathiratne, 2017b
– As – Cd – Pb trace concentrations	– *Danio rerio* ^m^	– Stunted growth – Developmental abnormalities (eye area↓, total body length↓, yolk sac area↑) – ALP^n^ and GPT^o^ activities ↑ – LDH^p^ and GOT^q^ activities ↓ – Overall number of proteins ↓	– Thomas et al., 2024
– As – Cd	– Etroplus suratensis – Anabas testudineus – Channa striata	– Metal accumulation (kidney, liver, gill and muscle)	– Perera et al., 2016
– Bulk-TiO_2_	– Oreochromis niloticus	– Liver damage indices ↑ – RBC DNA damage ↑ – Gill AChE^i^ activities ↑ – Liver AChE^i^ activities ↑	– Abegoda-Liyanage and Pathiratne, 2023 – De Silva and Pathiratne, 2023
– Nano-TiO_2_	– Oreochromis niloticus	– Erythrocytic DNA damage – RBC micronuclei ↑ – Necrosis (hepatic parenchyma and intestinal mucosa) – RBC counts, hemoglobin levels, total leucocyte counts and percent neutrophils in the peripheral blood ↑ – Depressed total phagocytic and myeloperoxidase activities of the blood ↓ – Serum lysozyme activities ↑ – Gill: Epithelial separation, mucous cell proliferation, hyperplasia and lamellae fusion – Hepatocytes: Vacuolations, pycnotic nuclei, apoptosis – Intestine: Eroded villi epithelium, mucous cells↓ and degeneration of mucosa – Liver AChE^i^ activities ↑	– Perera, and Pathiratne, 2014 – Abegoda-Liyanage and Pathiratne, 2023 – De Silva and Pathiratne, 2023
Different effluent types			
– Treated effluents of natural rubber processing industries	– Poecilia reticulata	– RBC abnormalities	– Karunarathne and Pathiratne, 2024
– Textile industry effluents	– Oreochromis niloticus	– AChE^i^ activities ↓ – EROD^c^ levels ↑ – RBC micronuclei and nuclear abnormalities ↑ (blebbed nuclei, notched nuclei, lobed nuclei, binucleated)	– Perera and Pathiratne, 2013
– Contaminated sediments containing water of maritime fisheries harbor	– Oreochromis niloticus	– Cumulative growth rates↓ (absolute growth rate, relative growth rate, and specific growth rate) – Gill: Lamellae-fusion and decreased inter-lamellar space – Liver: Extensive necrosis and hydropic vacuolation	– Gunawickrama et al., 2014

↑ indicate the increase, induced or elevated level of afore mentioned impacts; ↓ indicate the decrease, depressed, reduced, inhibited level of afore mentioned impacts.

^a^ Polycyclic aromatic hydrocarbons.

^b^ Sorbitol dehydrogenase.

^c^ Ethoxyresorufin-O-deethylase.

^d^ Glutathione S-transferase.

^e^ Fluorescent aromatic compounds.

^f^ Cholinesterase.

^g^ Insecticide.

^h^ Weedicide.

^i^ Acetylcholinesterase.

^j^ Bisphenol-A.

^k^ Juvenile.

^l^ Bisphenol-S.

^m^ Embryo.

^n^ Alkaline phosphatase.

^o^ Glutamic pyruvic transaminase.

^p^ Dehydrogenase.

^q^ Glutamic oxalacetic transaminase.

Among the most extensively studied contaminants are polycyclic aromatic hydrocarbons (PAHs) such as naphthalene, phenanthrene, pyrene, and benzo(a)pyrene. These compounds cause oxidative stress, enzymatic alterations, histological alterations, and genotoxicity in several freshwater species, including *O. niloticus*, *Oreochromis mossambicus, Rasbora caverii, Mugil cephalus*, *Lutjanus russellii*, *and Etroplus suratensis*. Documented effects include elevated hepatic enzyme activity (EROD and GST), increased biliary fluorescent aromatic compounds, depressed muscle cholinesterase (ChE) activity, and pronounced histopathological lesions in liver and gills, often accompanied by erythrocyte nuclear abnormalities such as micronuclei and nuclear buds (Wijeyaratne and Pathiratne, 2006; Pathiratne and Hemachandra, 2010; Pathiratne et al., 2010a; Hemachandra and Pathiratne, 2014; Ranasingha and Pathiratne, 2015). So, Sri Lankan freshwater fish exposed to PAHs show strong biomarker responses (EROD, GST, ChE), increased biliary PAH metabolites, and significant genotoxic and histopathological effects. These effects are consistently observed in polluted urban and coastal water bodies, highlighting the ecological risk of PAH contamination in Sri Lanka. Exposure to petroleum refinery wastewater and oil-contaminated water, both of which contain PAHs, causes genotoxic and behavioral changes, including sluggish movement and diverse nuclear deformities in erythrocytes of *O. niloticus*, *Trichogaster pectoralis*, and *Dawkinsia singhala*. Fish exposed to refinery wastewater showed bile fluorescence patterns indicative of exposure to naphthalene, phenanthrene, pyrene, and benzo(a)pyrene (Kanthi and Jayaweera, 2021; Weerakkodige et al., 2021).

Exposure to crude oil causes growth retardation and erythrocytic nuclear deformities (Gunawickrama et al., 2016). Juvenile *O. niloticus* was exposed for 16 weeks to sediments from the Galle fisheries harbor at two contamination levels, where T1: 1 unit-dose, T2: 3 unit-doses; 1 unit = 38 mL wet sediment. Growth rates in T1 and T2 were significantly lower than those in the controls (*p* < 0.05). Lamellae fusion and a decreased interlamellar space in the gills, as well as extensive necrosis and hydropic vacuolation in the liver, were observed. This conclusion suggests that chronic exposure to contaminated harbor sediment caused marked growth reduction and severe histopathological lesions in the gills and liver (Gunawickrama et al., 2014). Juveniles *O. niloticus* was exposed for 90 days to waterborne crude oil at 5 ppm and 25 ppm. Both exposure groups exhibited significantly reduced weight-based and length-based growth rates, as well as specific growth rates, compared to the controls (p < 0.05). Fish exposed to 25 ppm crude oil had significantly increased frequencies of micronuclei, nuclear buds, fragmented/apoptotic nuclei, and other ENA types in both peripheral blood and head kidney (*p* < 0.05). ENA frequencies were quantitatively higher in the head kidney than in the peripheral blood (Gunawickrama et al., 2016).

Different agrochemicals in lotic ecosystems make detrimental biological impacts on different indigenous fish species. Weedicide formulation, Gramoxone (Paraquat), two organophosphate insecticide formulations, Lebaycid (Fenthion) and Elsan (Phenthoate), cause significant AChE inhibition (*P* < 0.05) and gill histological changes, such as hyperplasia, hypertrophy, and enlargement of secondary lamellae, in *R. caverii* (Wijeratne and Pathiratne, 2006). Organophosphate and N-methyl carbamate pesticides inhibit acetylcholinesterase (AChE) activity in *G. ceylonensis*, *Devario malabaricus*, and *R. daniconius* (Sumith et al., 2012). In *Garra ceylonensis*, muscle acetylcholinesterase (AChE) activity showed up to 73% inhibition during the Yala season, which corresponds to the period of intensive cultivation. Eye AChE activity exhibited even greater sensitivity, with inhibition levels exceeding 70% in both Yala (76%) and Maha (72.5%) seasons. In *D. malabaricus*, brain AChE activity was inhibited by up to 47.8%, while muscle AChE activity showed inhibition up to 64.6% during the Yala season. Similarly, *Rasbora daniconius* displayed brain AChE inhibition reaching 60% and muscle AChE inhibition up to 56% during the same period. Pesticide concentrations and AChE inhibition were highest during the Yala season, which coincided with low-flow conditions and intensive pesticide use. *Devario malabaricus* and *R. daniconius* showed less dramatic AChE inhibition in eye tissues, suggesting the presence of some tissue-specific protective mechanisms (Sumith et al., 2012). Among agrochemical pollutants, carbosulfan and bispyribac-sodium lead to hepatic apoptosis and gill damage in *P. reticulata* and *A. parvus.* TUNEL staining revealed a significantly higher percentage of damaged nuclei; therefore, apoptotic cells in the liver of both *Aplocheilus parvus* in bispyribac-sodium exposure and *P. reticulata* in carbosulfan exposure at all but the lowest tested concentrations, compared to controls (ANOVA, df 5, 54, *P* < 0.005). For *A. parvus*, significant nuclear damage was observed at concentrations ≥0.1 mg/L bispyribac-sodium. For *P. reticulata*, all carbosulfan treatments resulted in significantly more liver damage than controls (Fernando et al., 2018).

Recent Sri Lankan studies have demonstrated that both Bisphenol A (BPA) and Bisphenol S (BPS) significantly disrupt the behavior, growth, survival, and reproductive health of juvenile zebrafish (*D. rerio*), with BPS often exhibiting effects comparable to or greater than those of BPA (Pathiraja and Rajapaksa, 2019; Senarath Pathirajage and Rajapaksa, 2024; Shanika and Rajapaksa, 2024; Shanika and Rajapaksa, 2025). Shanika and Rajapaksa (2025) exposed BPA and BPS at 50 μg/L for 60 days, Dulanthi and Rajapaksa (2023) at 10 and 100 μg/L for 35 days, and Senarath Pathirajage and Rajapaksa (2024) and Pathiraja and Rajapaksa (2019) at 1 and 10 μg/L for 60 days. Survival dropped to 56.6% (1 μg/L BPA) and 41.7% (10 μg/L BPA) vs. 90% in controls after 60 days (Pathiraja and Rajapaksa, 2019). Both BPA and BPS significantly increased not only mortality but also length, weight, and specific growth rate (*p* = 0.00) at 50 μg/L, compared to controls (*p* = 0.017) (Shanika and Rajapaksa, 2025). At 100 μg/L, both bisphenols significantly increased body weight increment (*p* < 0.05), but not body length or specific growth rate (Dulanthi and Rajapaksa, 2023). Both BPA and BPS significantly reduced the condition factor (*p* < 0.05), indicating poorer overall health (Dulanthi and Rajapaksa, 2023; Senarath Pathirajage and Rajapaksa, 2024). Both BPA and BPS induced a significant female-biased sex ratio, with BPS causing a more potent effect. Both bisphenols significantly reduced swimming speed (*p* = 0.001), leading to vacuolization and necrosis in the liver, as well as hyperplasia and mucous secretion in the gills (Senarath Pathirajage and Rajapaksa, 2024; Shanika and Rajapaksa, 2025). Both increased aggression (*p* = 0.00), with BPA showing a higher effect than BPS (*p* = 0.032) (Shanika and Rajapaksa, 2025). Histopathological alterations were observed in the gonads, indicating reproductive toxicity (Senarath Pathirajage and Rajapaksa, 2024). BPA exposure (10 μg/L) caused lipid accumulation in hepatocytes and degeneration of renal tubules (Pathiraja and Rajapaksa, 2019).

Pharmaceutical residues, such as acetaminophen (APAP), cause altered gill and liver histopathology, as well as behavioural changes in *O. niloticus* and *D. rerio* juveniles, which manifest as impaired feeding, altered swimming, and elevated vitellogenin expression, indicative of endocrine disruption. Both fish groups exposed to waterborne acetaminophen showed reduced physical avoidance responses, at 76.7% in treatment 1% and 68.7% in treatment 2, compared to the control fish at 87.3%. In addition, their mean ventilation rates were lower than those of the control group (Dissanayaka et al., 2024). Frequency of bi-nucleated, notched, lobed, and blebbed nuclei in APAP-exposed groups increased, with more pronounced effects at higher concentrations (Dissanayaka et al., 2024; Ishara et al., 2024; Rajapaksa and Ishara, 2025).

Microcystins, toxins produced by cyanobacterial blooms, induce similar hepatic and enzymatic disturbances in *O. niloticus*, including elevated serum SDH and GST activity, as well as inhibition of ChE. Fish sampled during dry periods showed a significant increase in serum SDH, indicating liver damage. Hepatic GST activity was reduced in fish with liver abnormalities, but in fish with apparently normal livers, GST activity was induced, reflecting exposure to microcystins and PAHs. Muscle ChE activities were significantly depressed in resident fish, indicating exposure to anticholinesterase substances, including microcystins and possibly PAHs (Pathiratne et al., 2010b).

Heavy metal accumulation in fish muscles has been reported in several lotic systems in Sri Lanka (Allinson et al., 2010; Perera et al., 2016). Allison et al. (2010) investigated that *O. niloticus* and *O. mossambicus* fish inhabiting Aruvi Aru, Kala Oya, Kirindi Oya, Ma Oya, Mahaweli, and Walawe River catchments are contaminated with metals Ca, Cu, Fe, K, Mg, Mn, Na, and Zn at varying levels. Although metal concentrations were below the WHO and FSANZ guideline values for fish, accumulated metal levels may still pose imperceptible biological impacts. Heavy metals (Cd, Cr, Cu, Pb, Zn, As) are widespread contaminants with well-documented neurotoxic, hematological, and histopathological effects. In *O. niloticus*, *E. suratensis*, *D. singhala*, and *P. reticulata*, exposure leads to reduced ChE activity, liver lesions, decreased red and white blood cell counts, and elevated mortality (Ruvinda and Pathiratne, 2018; Ruvinda and Pathiratne, 2020; Arambawatta-Lekamge et al., 2021; Dayananda and Liyanage, 2021; Gayashan et al., 2023). Fish from polluted sites, such as Kaduwela and Mattakkuliya, had significantly higher liver histopathological condition indices and erythrocytic nuclear abnormality frequencies than those from the less polluted site in Ruwanwella (*p* < 0.05). Water at the most polluted site (Mattakkuliya) had Cd, Cu, and Pb levels exceeding aquatic life protection criteria, with measured ranges: Cd (0.118–0.775 μg/L), Pb (0.078–5.34 μg/L), Cr (0.0016–3.50 μg/L), Cu (3.11–14.44 μg/L) (Ruvinda and Pathiratne, 2020). Laboratory exposure to polluted river water resulted in a 24% inhibition of brain cholinesterase (ChE) activity after 5 days (*p* < 0.05), indicating neurotoxicity (Ruvinda and Pathiratne, 2018). Even at trace levels in raw source water or water treatment effluents, these metals cause DNA strand breaks and micronuclei formation (Hemachandra and Pathiratne, 2017a). Similarly, trace metal mixtures have been shown to disrupt embryonic development in *D. rerio*, causing stunted growth and altered enzyme activities. Exposure to low concentrations of As, Cd, and Pb has been found to reduce the survival rate and hatchability of zebrafish embryos markedly (Thomas et al., 2024).

Emerging contaminants, such as titanium dioxide nanoparticles (TiO_2_ NPs), induce oxidative stress and extensive tissue injury in *O. niloticus*. Reported effects include DNA damage, hepatic necrosis, gill hyperplasia, and altered immune responses. Bulk TiO_2_ elicits similar though less pronounced biochemical alterations (Perera and Pathiratne, 2014; Abegoda-Liyanage and Pathiratne, 2023; De Silva and Pathiratne, 2023). Both nano- and bulk-TiO_2_ at 0.1 mg/L induced significant erythrocytic DNA damage after 7 days, as measured by the comet assay. At 14 days, DNA damage was observed at both 0.05 and 0.1 mg/L nano-TiO_2_ and at 0.1 mg/L bulk-TiO_2_. DNA damage was not fully repaired after a 7-day recovery period, indicating persistent genotoxic effects (Abegoda-Liyanage and Pathiratne, 2023; De Silva and Pathiratne, 2023).

Effluents from natural rubber processing industries induce erythrocytic deformities in *Poecilia reticulata*, highlighting the cellular-level toxicity of industrial discharges. Even after wetland treatment, effluents retained cytogenotoxic contaminants, as evidenced by persistent erythrocytic abnormalities in exposed fish. The study highlights that current effluent treatments are insufficient to eliminate cytogenotoxic hazards, emphasizing the need for improved waste management to protect aquatic life (Karunarathne and Pathiratne, 2024). Effluents from the textile industry are genotoxic, depressing AChE activity and inducing erythrocyte nuclear abnormalities in *O. niloticus*. Fish exposed to undiluted and diluted textile effluents showed up to 40% reduction in brain AChE activity compared to controls, indicating the presence of neurotoxic substances. Frequencies of erythrocytic micronuclei and nuclear alterations increased up to 9-fold in exposed fish, indicating a strong genotoxic effect. Hepatic EROD activity, a marker for exposure to certain organic pollutants, was induced up to 23-fold in exposed fish, suggesting the presence of CYP1A-inducing and potentially genotoxic chemicals (Perera and Pathiratne, 2013). Contaminated sediments from harbour areas lead to growth inhibition and severe gill and hepatic degeneration (Gunawickrama et al., 2014).

Research on the impact of pollution mixtures on Sri Lankan fish populations reveals the behavioral, morphological, and physiological effects of pollution mixtures on fish (Table 1). However, many novel chemicals have been tested under laboratory exposure systems, which hinder the real-world effects. Organic and inorganic pollutants, as well as novel xenobiotics, can have synergistic and antagonistic effects that are far more damaging than the effects of individual components under climate change.

### 3.4 Climate change and pollution interactions

**FIGURE 3:**
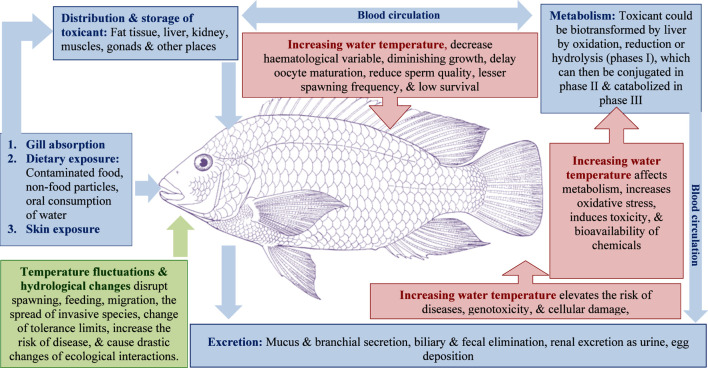
Mechanism diagram of fish ecophysiological responses under the co-action of pollution and climate.

Climate change impacts on lotic ecosystems were a growing concern at the beginning of the millennium. Rivers and streams are susceptible to alterations of thermal regimes and intensification of hydrological cycles, leading to shifted phenology, reduced dissolved oxygen, and the loss of cold-water specialist species (Nijssen et al., 2001; Ficke et al., 2007; Heino et al., 2009; Palmer et al., 2008; Woodward et al., 2010). Pollution alone has well-documented consequences, including immune suppression, metabolic disruption, damage to gills and other internal tissues, and increased susceptibility to disease (Ahmed et al., 2022). When coupled with climate stressors (As illustrated in Figure 3) pollutant toxicity can become synergistic, amplifying physiological disturbances, altering reproductive behavior, and threatening long-term population stability (Ramirez-Duarte et al., 2025).

Increased water temperatures and altered flow regimes impose physiological stress on fish populations (Huang et al., 2021; Sims, 2025). These climatic shifts also increase the toxicity and bioavailability of pollutants, such as pesticides, heavy metals, and nutrients, thereby exacerbating their effects on fish survival and reproduction (Malik et al., 2020; Ramirez-Duarte et al., 2025; Sims, 2025). Raised temperatures like 2 °C–3 °C above historical norms disrupt endocrine signaling, decrease cellular energy status, delay oocyte maturation, reduce sperm quality, and shorten spawning windows, leading to lesser spawning frequency and fry survival in species like *Salmo trutta*, *Labeo rohita*, *Catla, Spirinchus thaleichthys*, *Hypomesus transpacificus* and*, Salvelinus fontinalis* (Jeffries et al., 2016; Hampuwo et al., 2025; Patel, 2025; Sims, 2025). In brown trout, a 1.5 °C–2 °C increase in water temperature can reduce suitable habitat by up to 64% by 2080, leading to increased physiological stress, diminished growth, and a higher risk of diseases such as proliferative kidney disease (Borgwardt et al., 2020; Hampuwo et al., 2025).

*Carassius auratus* exposed to a pesticide cocktail at 32 °C (compared to 22 °C) exhibited higher genotoxic effects, including a higher micronuclei rate and irreversible cellular damage, such as apoptosis and necrosis, in the gills and liver, along with decreased reproductive indices (Jacquin et al., 2019). In a study using *S. trutta*, the number of genes affected by pesticides was greater at 15 °C than at 12 °C, indicating temperature-dependent toxicity (Voisin et al., 2025). *Oreochromis niloticus* exposed to cadmium (0.5 mg/L) at 32 °C exhibited significantly elevated oxidative stress enzyme activity and decreased haematological variables compared to lower temperatures, indicating enhanced cadmium toxicity (Abdel-Tawwab and Wafeek, 2017).

Rising temperatures and changing precipitation patterns are also modifying hydrological cycles and habitat suitability in tropical river systems (Siddha and Sahu, 2022; Sadhwani et al., 2023). Tropical freshwater fishes, which already live near their upper thermal limits, are susceptible to such changes (Barbarossa et al., 2021). For instance, flow depletion, combined with nutrient pollution, resulted in low diversity, impaired biomass, and altered macroinvertebrate and fish communities (Barletta and Lima, 2019; Chen et al., 2025). More frequent hot days and reduced thermal refugia narrow their tolerance ranges, while irregular rainfall and hydrological extremes disrupt spawning, feeding, and migration (Barbarossa et al., 2021).

Recent research on climate predictions has inferred that Sri Lankan vertebrates in high-elevation areas are declining in faster rates (Wijerathne et al., 2025). Even though this study did not consider fish in its geospatial predictions, the ecosystems with the highest endemic and indigenous fish diversity have been recorded of prone to being impacted by climate change scenarios in the future, forcing irrevocable stress on fish. Climate change and pollution are intensifying threats to freshwater fishes in Sri Lanka’s lotic ecosystems, with combined impacts on habitat quality, fish health, and biodiversity. These effects are particularly severe in Sri Lanka’s wet zone, where endemic species with restricted ranges face an increased risk of extinction (Priyadarshana et al., 2024).

Pollution further compounds these climate-driven impacts. Nutrient enrichment leads to eutrophication and oxygen depletion, while chemical contaminants, including heavy metals, agrochemicals, and microplastics, create chronic toxic stress in fish populations (Cera et al., 2020; Batuwita et al., 2022; Bonet et al., 2023). Rising temperatures and altered hydrology increase the bioavailability and mobility of these contaminants, enhancing methylmercury production and accumulation in fish tissues, with potential ecological and food safety implications (Bonet et al., 2023). Increased water stratification and altered flow regimes from sea-ice melt or reservoir flooding stimulate microbial methylation of mercury, especially in surface waters rich in organic matter. For example, in Arctic estuaries, flooding can increase MeHg inputs by 25%–200%, and climate-driven stratification further elevates MeHg levels in plankton and fish (Schartup et al., 2015). Reduced oxygen concentrations from warming, combined with nutrient-driven hypoxia, heighten metabolic stress and mortality risk (Barbarossa et al., 2021; Batuwita et al., 2022). Furthermore, hydrological extremes such as intense rainfall and droughts alter contaminant loading: floods deliver pulses of pesticides, plastics, and sediments into rivers, while droughts concentrate pollutants and increase exposure intensity (Bonet et al., 2023).

Climate warming also facilitates the spread of invasive fishes, which can alter food webs and intensify the effects of pollutants on native and endemic species (Carosi et al., 2023). Comparative modeling from the Mekong Basin demonstrates that climate drivers often outweigh local habitat factors in shaping future fish distributions, suggesting similar outcomes for Sri Lanka’s tropical river systems (Nuon et al., 2024). Collectively, multiple lines of evidence indicate that climate change and pollution interact synergistically to restructure freshwater fish communities, threatening Sri Lanka’s endemic biodiversity. These findings underscore the need for integrated management approaches that address both global drivers, such as climate change, and local pressures, including pollution and habitat degradation (Lange et al., 2018; Barbarossa et al., 2021; Bonet et al., 2023; Carosi et al., 2023). However, current research in Sri Lanka is insufficient to demonstrate the link between climate change and the impacts of water pollutants on fish (Surasinghe et al., 2019).

## 4 Discussion

Sri Lanka has 224.35 km^2^ of rivers and riverine forest areas, which support diverse aquatic floral and faunal communities (Dananjaya and Wijeratne, 2017). The geographical location of Sri Lanka in the Oriental region makes it a climate hazard-prone area, similar to other South Asian countries (Amarnath et al., 2017). Available geospatial and climate data extrapolate the range of climate hazards, including floods, droughts, sea-level rise, and extreme rainfall, in Sri Lanka for the foreseeable future (Climate Change Secretariat, 2016; Amarnath et al., 2017; Nisansala et al., 2020; Alahacoon and Edirisinghe, 2021; Samaraweera et al., 2024; De Silva and Ekanayake, 2025). There is limited integration between climate-related stressors, such as elevated temperatures, altered hydrology, and pollution toxicodynamics, in local studies. Nevertheless, such straightforward interactions have been shown elsewhere, emphasizing the intensification of pollutant bioavailability and toxicity due to climate variability (Barbarossa et al., 2021; Bonet et al., 2023). No available studies directly investigate how acute temperature changes interact with pollutants to affect freshwater fish in Sri Lanka. One apparent reason is the unavailability of data related to freshwater temperature fluctuations and hydrological changes. Wijeratne et al. (2025) predict that climate-related issues may affect the southwestern and central mountain regions of the country, where the most endemic and sensitive species are found.

Urbanization, increased settlements, industrial effluents, agricultural inputs, and accidental releases bring a mixture of pollutants to lotic ecosystems. However, the inherent capacity of freshwater fish to dilute and detoxify substances discharged into their environment and to recover their original nutrient and oxygen levels alleviates the detrimental impacts on freshwater fish inhabitants in rivers. Nevertheless, many lotic systems have become overloaded with pollutants and may lack the capacity to recover from increasing levels of pollution due to unintended climate influences. The high specific heat capacity of water prevents rapid temperature rises; however, elevated ambient temperatures can still accelerate metabolic activity and increase pollutant uptake in aquatic organisms. Moreover, higher temperatures can enhance the solubility of specific contaminants that were previously in solid or particulate form, facilitating their dissolution into the water column and thereby increasing their bioavailability and toxicity to aquatic life. Concurrently, hydrological alterations, such as reduced dilution during dry, low-flow periods or intense rainfall and runoff events, can concentrate or mobilize pollutants, amplifying their ecological impacts on freshwater systems (Sunardi and Wiegleb, 2016; Bonet et al., 2023).

Across studies conducted in Sri Lanka, *O. niloticus* (Nile tilapia) emerges as the most extensively used fish species for ecotoxicological research and biomonitoring. Its broad tolerance to physicochemical stressors, easy maintenance in laboratory conditions, and rapid growth make it an attractive sentinel organism (Pathiratne and Hemachandra, 2010; De Silva and Pathiratne, 2023; Ruvinda and Pathiratne, 2018; Pathiratne et al., 2009). Numerous investigations employing *O. niloticus* have explored pollutant-induced effects, including exposure to polycyclic aromatic hydrocarbons (PAHs), heavy metals, textile effluents, petroleum hydrocarbons, nanoparticles, and pharmaceuticals. These studies have consistently reported biomarker responses such as inhibition of acetylcholinesterase (AChE) activity, altered ethoxyresorufin O-deethylase (EROD) and glutathione S-transferase (GST) levels, erythrocytic nuclear abnormalities, and histopathological lesions in gill and liver tissues (Pathiratne et al., 2010b; Perera and Pathiratne, 2013; Gunawickrama et al., 2014; Abegoda-Liyanage and Pathiratne, 2023). The presence of Bisphenol A (BPA), a potent endocrine-disrupting compound with estrogenic activity, has been documented in Bolgoda Lake and its associated lotic ecosystem, which receives leachate from a solid waste open dump. Ecological risk assessments revealed that *O. mossambicus* and *Ctenopharyngodon idella* experience high acute risk during the wet season and medium acute risk during the dry period (Inam et al., 2015; Luo et al., 2019; Matharage et al., 2021). A similar study conducted in Kerawalapitiya, where raw leachate from a municipal solid waste dump contaminates nearby surface waters, also detected BPA. The corresponding risk assessments revealed that *O. mossambicus* and *Etroplus maculatus* were exposed to high acute risk during the wet season and medium acute risk during the dry season (Liu et al., 2017; Shehab et al., 2020; Matharage et al., 2024). These studies reveal the presence of BPA and its associated risks but lack an exploration of sublethal or physiological effects on exposed fish. The interplay between the BPA and climate change scenarios must be addressed to prevent health impairments in fish and humans.

To date, no published research from Sri Lanka has directly examined the impact of rare earth elements (REEs) on freshwater fish as pollutants. Studies in the country have assessed the accumulation and effects of heavy metals such as cadmium, arsenic, lead, mercury, chromium, copper, and zinc in freshwater fish and sediments (Hemachandra and Pathiratne, 2017a; Ruvinda and Pathiratne, 2018; Ruvinda and Pathiratne, 2020; Arambawatta-Lekamge et al., 2021; Dayananda and Liyanage, 2021; Gayashan et al., 2023; Thomas et al., 2024). These studies highlight the significant bioaccumulation of traditional heavy metals in fish tissues, which sometimes exceed international safety standards, and raise concerns about chronic health risks for both fish and humans consuming them. However, none of these studies include rare earth elements (such as lanthanum, cerium, neodymium, or gadolinium) in their analyses.

While several investigations have assessed heavy metal accumulation in freshwater species, most have been limited to quantification rather than evaluating the biological impact of these metals on the species. A study examined the levels of Pb, Cd, Cr, Cu, and Zn in *L. rohita*, *O. mossambicus*, *O. niloticus*, *E. suratensis*, *Heteropneustes fossilis*, *Oligolepis acutipennis*, and *Puntius dorsalis* from the Minneriya, Parakrama Samudraya, and Kaudulla reservoirs (Wijesinghe et al., 2018). Although the study identified *O. niloticus*, *E. suratensis*, and *O. acutipennis* as particularly vulnerable to bioaccumulation in head, muscle, and skin tissues, it did not evaluate associated physiological or pathological alterations. Similarly, cadmium accumulation in the kidney tissues of *O. niloticus* and *Ompok bimaculatus* from the Padaviya Reservoir was reported, indicating a strong tendency for renal bioaccumulation (Weerasekara et al., 2020). However, data on organ-level toxicity or histopathological effects remain unavailable. In edible muscle tissues, Cd levels reached 0.1 mg/kg, while As levels were below 0.05 mg/kg on a wet weight basis. Earlier, essential and trace metals such as Ca, Cu, Fe, K, Mg, Mn, Na, and Zn in *O. mossambicus* and *O. niloticus* from several Sri Lankan rivers, including Aruvi Aru, Kala Oya, Kirindi Oya, Ma Oya, Mahaweli, and Walawe, were found (Allinson et al., 2010). Additionally, Pb, Cd, Cr, Cu, and Zn were detected in muscle, gill, and liver tissues of *Mystus gulio* inhabiting Bolgoda Lake (Senarathne and Pathiratne, 2010). ‘More recently, *E. suratensis* from the Nikawewa and Mahakandarawa tanks contained Pb and Cr concentrations exceeding the Maximum Permissible Limits (MPL) in muscle tissues (Perera et al., 2024). Published studies related to fish have indicated the risk of heavy metals in lotic water; however, their interaction with novel pollution sources, such as microplastics, pharmaceuticals, and climate-related changes in water quality, remains to be studied.

Although these studies demonstrate widespread metal bioaccumulation, they collectively reveal a critical knowledge gap regarding the physiological and histopathological consequences of such contamination. Prior research has established that heavy metal exposure can inhibit brain cholinesterase (ChE) activity, increase the frequency of erythrocytic nuclear abnormalities (micronuclei, nuclear buds, binuclei, and notched nuclei), elevate histopathological indices, induce liver lesions, and elevate mortality rates in fish (Ruvinda and Pathiratne, 2018; Ruvinda and Pathiratne, 2020; Arambawatta-Lekamge et al., 2021; Dayananda and Liyanage, 2021). However, these effects have not yet been systematically investigated within the context of Sri Lanka’s inland freshwater lotic ecosystems.

While *O. niloticus* dominates the literature, fewer studies have incorporated indigenous or endemic freshwater fishes, such as *G. ceylonensis*, *R. daniconius*, *D. malabaricus*, and *Puntius titteya*. These native species were primarily used to assess the impacts of agrochemical or pesticide exposure on neurotoxicity markers, such as AChE inhibition (Sumith et al., 2012; Ranatunga and Abeyrathne, 2019). However, the limited application of such species constrains the ecological representativeness of Sri Lankan biomonitoring, since introduced tilapia may not accurately reflect the sensitivity of native taxa that have evolved under distinct local environmental pressures. Future studies should therefore expand biomarker and physiological assessments in endemic fishes to better understand their pollutant tolerance thresholds and conservation needs.

In terms of biomarker scope, genetic and molecular indicators remain underutilized. Although DNA damage in erythrocytes of *O. niloticus* has been detected following exposure to TiO_2_ nanoparticles and bulk TiO_2_, and DNA strand breaks due to exposure of raw river water and water treatment waste (effluents) were detected using the Alkaline *in vivo* comet assay (Abegoda-Liyanage and Pathiratne, 2023; Hemachandra and Pathiratne, 2017a). But comprehensive analyses involving molecular markers, gene expression, or genomic-level responses have not been reported for Sri Lankan freshwater fish. This represents a significant research gap, as such genetic biomarkers can provide early warning evidence of pollutant-induced stress and adaptation mechanisms. Similarly, oxidative stress parameters, such as catalase (CAT), superoxide dismutase (SOD), and lipid peroxidation (L) levels, have rarely been quantified in Sri Lankan contexts, despite strong international evidence linking these biomarkers to contaminant-induced oxidative damage (Subaramaniyam et al., 2023). Most local studies focus on enzyme inhibition (AChE, EROD, GST) and cytogenetic abnormalities rather than antioxidant defense mechanisms. Integrating oxidative stress assays could yield more mechanistic insights into pollutant toxicity under tropical environmental conditions.

Moreover, the endocrine-disrupting potential of pollutants remains scarcely investigated. Only a few studies have addressed estrogenic compounds, such as Bisphenol A (BPA) and Bisphenol S (BPS), using *D. rerio* juveniles, revealing female-biased sex ratios, altered gonadal histopathology, and behavioral disruptions (Pathiraja and Rajapaksa, 2019; Shanika and Rajapaksa, 2024). Despite the detection of BPA in Sri Lankan aquatic systems such as Bolgoda Lake and Kerawalapitiya (Matharage et al., 2021; Matharage et al., 2024), there are no published endocrine biomarker studies involving native or endemic fish species. Given the widespread release of pharmaceuticals and personal care products (Guruge et al., 2019), endocrine disruption represents a critical but overlooked threat that warrants urgent research attention.

Although changes in sex ratios have been observed in freshwater fish (*D. rerio*), there is no evidence of studies monitoring how these alterations influence breeding patterns (Pathiraja and Rajapaksa, 2019). There is no evidence of studies monitoring how these alterations influence breeding patterns and, consequently, affect community-level interactions within freshwater lotic ecosystems in Sri Lanka. While Sri Lankan studies confirm the presence and impact of pollutant mixtures, primarily heavy metals, pesticides, and organic contaminants, most focus on bioaccumulation and health risk rather than experimental assessment of combined toxicological effects or interactive mechanisms. There is limited research on how these mixtures interact synergistically or antagonistically in local fish species.

Research interest in microplastic pollution within Sri Lanka has been relatively limited, gaining momentum only after the X-Press Pearl maritime disaster. Even then, most microplastic studies have focused on marine environments, while investigations in inland freshwater systems remain scarce (Jayasiri et al., 2024; Kapukotuwa et al., 2025). Notably, no studies have yet examined the toxicological or physiological effects of microplastics on freshwater fish species inhabited in lotic ecosystems in Sri Lanka, highlighting a significant research void. The need to consider the impacts of microplastics under different environmental conditions, multiple stressors, and intrinsic factors of fish has been extensively discussed and emphasized (Parker et al., 2021). The unraveling the reproductive and transgenerational consequences of MNP exposure presents significant analytical challenges (Yi et al., 2024). Microplastics pose a significant ecological hazard in freshwater systems, serving as vectors for hazardous contaminants, including persistent organic pollutants (POPs) and heavy metals (Wu et al., 2025). Further, research is needed to understand the community and ecosystem-level impacts of microplastics in aquatic habitats (Parker et al., 2021; Parker et al., 2024; Lebepe et al., 2025). Spatial and temporal variations of microplastics were investigated, and it was suggested that variations in microplastic concentration may correlate with precipitation and stormwater runoff (Talbot and Chang, 2022). In addition to microplastics, contamination from pharmaceutical and personal care products (PPCPs) has emerged as a growing concern (Sirimanna and Nadarajah, 2025). Findings by Guruge et al. (2019) indicate that hospital effluents, household wastewater, and aquaculture activities contribute substantially to PPCP inputs into Sri Lankan waterways. Despite this, information regarding their ecological and physiological impacts on aquatic fauna, particularly freshwater fish, remains absent. These studies indicated that climate change scenarios may have a significant impact on the spatial and temporal variations of aquatic pollutant loads in the future.

## 5 Conclusion

Sri Lanka, designated alongside the Western Ghats as a global biodiversity hotspot, harbors a rich but fragile freshwater biodiversity, particularly within its lotic ecosystems. Despite this high level of endemism, these habitats are facing accelerated degradation of freshwater quality due to rapid urbanization, industrial discharge, and high population density (Goonatilake, 2012; Surasinghe et al., 2019). The current scientific literature indicates that traditional ecotoxicology is insufficient and fails to account for the ‘cocktail effect’ of pollutant mixtures combined with the thermal and hydrological stressors of climate change. The lack of data regarding how these combined stressors affect indigenous fish species represents a critical vulnerability in Sri Lanka’s conservation strategy. The synthesis of research data highlights three significant and dangerous synergies: 1. Fish are exposed to complex mixtures of organic and inorganic xenobiotics, agrochemicals, heavy metals, microplastics, and pharmaceuticals simultaneously; 2. Climate variables in the country, including rising water temperatures, altered flow rates, often ameliorate toxicity; 3. Toxicity data relies on hardy, non-native model species like Tilapia or Zebra fish, which do not reflect the sensitivity of Sri Lankan endemics such as *Esomus, Pethiya, Puntius, Devario, Dawkinsia, Rasbora, Garra, etc.* To address these challenges, intense action and policy reforms are proposed, aligned with the National Biodiversity Strategic Action Plan (MoMDandE, 2016) and National Environmental Act mandates. Legislative reform must regulate the cumulative load of pollutants from multiple sources, not just individual factory limits. Traditional chemical monitoring must be transformed into biological-effect monitoring using biomarkers (e.g., histological changes, enzyme activity, genetic damage) in indigenous fish during Environmental Impact Assessments (EIAs). Monitoring must assess the interaction between rising water temperatures and chemical toxicity. Identification and establishment of strictly protected upper-catchment areas with low pollution and stable water temperatures. These protected ecosystems may serve as “genetic arks” for endemic fish as recommended in the sixth National Report to the Convention on Biological Diversity (MoMDandE, 2019). Allocate specific funding for novel technological tools, such as mesocosm experiments—semi-natural outdoor experiments that simulate real-world conditions rather than sterile lab tests. Ultimately, establishing multi-stressor experimental frameworks that integrate xenobiotic exposure with consideration of climate variables could enhance predictions of future impacts on Sri Lanka’s freshwater biodiversity. Government and private-sector collaborative research should prioritize the ecological impacts of pollutant mixtures under climate variabilities, with robust national policy frameworks critical to mitigating the long-term effects on freshwater fishes in lotic ecosystems.
